# Preserving Eggs

**Published:** 1872-01

**Authors:** 


### PRESERVING EGGS.

Fresh eggs are a very great comfort; they are healthful and nutritious. The only way to have them really fresh all the year round is to arrange to have chickens hatched in the first week of every month in the year. One of the next best plans of having eggs, almost as good as new, from November to April, is to adopt the advice of a Herkimer County farmer, namely: —

A piece of lime, as large as a quart dipper, is put into five gallons of water, and salt added until an egg will float. This is strained and put into a clean keg, into which a loose head is made to fit easily; a knob is fitted to the head for a handle. The eggs are put, as they are gathered, into the liquid, and the loose head placed on them to keep them below the surface. The keg should be kept in a cool place in the cellar. The liquor will not freeze except at a lower temperature than freezing point.

